# Niche dimensions in soil oribatid mite community assembly under native and introduced tree species

**DOI:** 10.1002/ece3.11431

**Published:** 2024-05-20

**Authors:** Johanna Elisabeth Noske, Jing‐Zhong Lu, Ina Schaefer, Mark Maraun, Stefan Scheu, Ting‐Wen Chen

**Affiliations:** ^1^ J. F. Blumenbach Institute of Zoology and Anthropology University of Göttingen Göttingen Germany; ^2^ Senckenberg Biodiversity Climate Research Center Frankfurt am Main Germany; ^3^ Loewe Center for Translational Biodiversity Genomics (LOEWE‐TBG) Frankfurt am Main Germany; ^4^ Centre of Biodiversity and Sustainable Land Use University of Göttingen Göttingen Germany

**Keywords:** acari, beech, douglas fir, environmental filtering, niche partitioning, phylogenetic diversity

## Abstract

Forest soils are a critical component of terrestrial ecosystems and host a large number of animal decomposer species. One diverse and abundant decomposer taxon is oribatid mites (Acari: Oribatida), whose species composition varies with forest type and tree species composition. We used functional traits that indicate different niche dimensions, to infer assembly processes of oribatid mite communities in monocultures and mixed forests of native and introduced tree species. We found that coexisting species differed more in the resource‐related niche dimension, i.e., reproductive mode and trophic guild, than in the morphological dimension, e.g., body length and width, sclerotization and concealability. These results suggest that both filtering and partitioning processes structure oribatid mite communities. In native European beech forests, but not in non‐native Douglas fir forests, oribatid mites were mainly structured by filtering processes acting via traits related both to environmental tolerance and to resources. Furthermore, oribatid mite trait diversity, but not phylogenetic diversity, differed significantly between monocultures and mixed forests, demonstrating that multidimensional diversity indices provide additional information on soil biodiversity. Overall, the study provides evidence that traits representing different niche dimensions need to be considered for understanding assembly processes in soil animal communities and thereby soil biodiversity.

## Graphical abstract

Using functional traits, our study showed that oribatid mite communities were structured by both filtering and partitioning processes. Coexisting species differed more in resource‐related (α‐niche) traits than in morphological (β‐niche) traits, and were driven more by tree species identity (beech vs. conifer) than by forest type (monoculture vs. mixed forest).

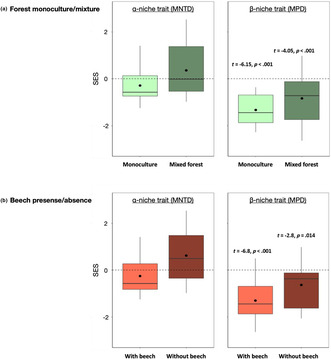

## 1 INTRODUCTION

Forests in Central Europe have been managed for centuries, altering the diversity of flora and fauna (Erdmann et al., 2012; Seibold et al., 2019). Climate change and extreme weather events are likely to increase in the future. It is therefore essential to increase the resistance and resilience of forests to disturbances in order to maintain ecosystem functions and services. One‐third of Germany is covered by forests, most of which are monocultures. Stand diversification through mixed forests has been proposed to increase ecosystem functions and services (Ammer, 2019; Schuldt et al., 2020). The most common naturally occurring tree species is European beech (*Fagus sylvatica* L.). However, Norway spruce (*Picea abies* (l.) h. karst.) has been introduced into lowland ecosystems where it is often planted in monocultures (Ellenberg, 1996). In recent years, spruce monocultures have been facing increasing damage from drought and insect outbreaks (Boczoń et al., 2018; Hlásny et al., 2021; Hlásny & Turčáni, 2013). As a more drought‐resistant alternative to Norway spruce, the integration of the non‐native Douglas fir (*Pseudotsuga menziesii* (mirbel) franco) into European forests is receiving increasing attention (Bolte et al., 2009; Schmid et al., 2014).

The conversion to mixed forests with different combinations of native and introduced tree species affects not only plant communities but also soil microorganisms and soil animals (Leidinger et al., 2021; Zaitsev et al., 2014). Both microorganisms and animals in soil are critical components of forest ecosystems, providing a range of functions such as organic matter decomposition and nutrient cycling, which consequently affect tree performance. Oribatid mites (Acari: Oribatida) are a major component of the soil animal community reaching particularly high densities in forests including those in the temperate zone (Maraun & Scheu, 2000). Their density and community composition have been shown to vary with forest type (i.e., deciduous vs. coniferous) rather than with the number of tree species (Erdmann et al., 2012; Lu, 2021; Lu, Bluhm, et al., 2022). Mixed forests are likely to provide greater resource and microhabitat diversity, allowing species to occupy different positions in niche space (Chesson, 2000; Nielsen et al., 2010; Scott & Baer, 2019).

Both resource‐based niche partitioning and environmental filtering have been shown to structure oribatid mite communities (Ingimarsdóttir et al., 2012; Maaß et al., 2015; Magilton et al., 2019). Morphological traits, reproductive mode, and trophic guild have been used to infer community assembly processes (Magilton et al., 2019; Maraun et al., 2019, 2023; Salazar‐Fillippo et al., 2023). Oribatid mites are diverse in their traits such as body form and size, sclerotization, and concealability (e.g., presence of pteromorphs). Based on these traits, oribatid mite species have been classified into ecomorphological groups that reflect the ecological niche of different species in soil (Salazar‐Fillippo et al., 2023). These traits changed little over hundreds of millions of years, suggesting that the niches of oribatid mites in soil were rather stable over time (Schaefer & Caruso, 2019; Sidorchuk, 2018). Phylogenetic and trait information can provide a mechanistic understanding of how species from the regional species pool assemble into local communities (Chen, 2018; Xie et al., 2022). However, studies on oribatid mite communities from both ecological and evolutionary perspectives are lacking.

Here, we classified oribatid mite traits into α‐ and β‐niche dimensions to disentangle the processes contributing to the assembly of communities (Ackerly & Cornwell, 2007). Generally, α‐niche traits are related to resource use and β‐niche traits to coping with abiotic factors. From an ecological perspective, coexisting species are likely to partition their α‐niche to avoid competition, thus showing a pattern of overdispersion; by contrast, β‐niche traits of coexisting species are expected to be similar, resulting in a clustering pattern reflecting environmental filtering (Silvertown et al., 2006). Evolutionarily, α‐niche traits tend to be labile, whereas β‐niche traits typically are phylogenetically conserved (Ackerly et al., 2006; Silvertown et al., 2006; but see Saban et al., 2023). Therefore, α‐ and β‐niche traits are likely to differ both ecologically and evolutionarily between coexisting species. Although this classification is informative for understanding community assembly processes, it has not yet been applied to soil animals.

In this study, we used the α‐ and β‐niche trait concept to infer assembly processes of oribatid mite communities inhabiting different forests, i.e., monocultures vs. mixed forests and presence of beech or coniferous species. We used distance‐based metrics to quantify phylogenetic and trait distances between pairs of species in a community to infer community assembly processes (Swenson, 2014, 2019). We tested the following hypotheses:

- Phylogenetic and trait diversity represented by the observed mean pairwise distances are greater in oribatid mite communities in mixed forests than in monocultures.
- Both niche partitioning and environmental filtering operate in oribatid mite communities, with α‐niche traits showing overdispersion (more distant relatedness than expected by random assembly processes) and β‐niche traits showing clustering patterns (closer relatedness than expected).
- Oribatid mite community assembly processes are driven more by tree species identity (beech vs. conifer) than by forest type (monoculture vs. mixed forest).

## 2 MATERIALS AND METHODS

### 2.1 Study sites and species collection

Eight study sites were established in the state of Lower Saxony, Germany, in 2017 (Figure S1). Five forest types were selected at each site. The five forest types included pure European beech (*F. sylvatica*), pure Norway spruce (*P. abies*), pure Douglas fir (*P. menziesii*), mixed beech and Douglas fir, and mixed beech and Norway spruce. The trees were on average at least 50 years old. A total of 40 plots (50 m × 50 m) were established with a minimum distance between plots of 76 m and between sites of 4.6 km (Ammer et al., 2020; Foltran et al., 2020; Lu & Scheu, 2021).

At each of the 40 forest plots, one soil sample (ø 5 cm, 10 cm depth) was taken between November 2017 and January 2018. Animals were extracted by heat (Kempson et al., 1963), collected in 50% diethylene glycol, and then stored in 70% ethanol until further analysis (Lu, 2021). Of the 71 oribatid mite species found, 14 species occurred in single plots and were excluded from analyses (Lu, Bluhm, et al., 2022), as rare species are unlikely to contribute much to the general patterns that structure community assembly processes. We considered the remaining 57 species occurring in more than one plot as the species pool from which local communities are assembled.

### 2.2 Phylogenetic reconstruction of the oribatid mite species pool

We downloaded oribatid mite 18S rDNA sequences from GenBank for 26 species in the species pool (Data S1). We further sequenced 18S rDNA of nine species, i.e., *Cultroribula bicultrata*, *Chamobates borealis*, *Porobelba spinosa*, *Euzetes globulus*, *Oppiella falcata*, *Oribatella quadricornuta*, *Eupelops torulosus*, *Steganacarus striculus*, and *Scheloribates initialis* (Weigmann, 2006; GenBank Accession Numbers OR770591‐OR770599). Species were collected from litter samples from the study plots in March 2022 and identified to species level using Weigmann (2006). We extracted genomic DNA from one to ten individuals of each species, depending on body size, using the DNeasy Blood & Tissue Kit (Qiagen, Hilden, Germany) according to the manufacturer's protocol and preserved the vouchers. We amplified the 18S rDNA region in three segments with the following primer pairs: 1st segment: forward (5'‐TACCTGGTTGATCCTGCCAG‐3′) and 614r (5'TCCAACTACGAGCTTTTTAACC‐3′); 2nd segment: 554f (5'‐AAGTCTGGTGCCAGCAGCCGC‐3′) and 1282r (5'‐TCACT CCACCAACTAAGAACGGC‐3′); 3rd segment: 1150f (5'‐ATTGACGGAAGGGCACCACCAG‐3′) and reverse (5'‐TAATGATCCTTCCGCAGGTTCAC‐3′) (Domes et al., 2007). PCR reactions were performed in 25 μL volumes containing 12.5 μL SuperHot Taq Mastermix (Genaxxon Bioscience GmbH, Ulm, Germany), 1.5 μL MgCl_2_ (25 mM), 1 μL of each primer (10 pM), 1 μL BSA, 4 μL ultrapure water, and 4 μL template DNA. The PCR protocol consisted of an initial activation step at 95°C for 15 min, 35 amplification cycles of denaturation at 95°C for 15 s, annealing at 57°C (1st and 3rd primer pair) and 59°C (2nd primer pair) for 60 s and elongation at 72°C for 60 s, followed by a final elongation at 72°C for 10 min. PCR products were visualized on a 1% agarose gel. Positive products were purified using the PCR/DNA Purification Mini Spin Column Kit (Genaxxon Bioscience GmbH, Ulm, Germany) and sequenced at Microsynth Seqlab GmbH (Göttingen, Germany). In total, we obtained 35 of the 57 species in the species pool for phylogenetic reconstruction.

We used two Palaeosomata taxa, *Palaeacarus hystricinus* Trägårdh and *Stomacarus ligamentifer* Hammer, as outgroups. We aligned the sequences using the ClustalW multiple alignment algorithm in BioEdit with a gap opening penalty = 10 and a gap extension penalty = 0.1 (Hall, 1999; Thompson et al., 1994) and trimmed the alignment to a final length of 1858 bp. Based on the initial neighbor joining tree (Gascuel, 1997), we selected the best‐fitting model of sequence evolution and then reconstructed a maximum likelihood phylogram with 500 bootstraps (Figure S2) using the pml algorithm in the *R* packages *ape* (Paradis & Schliep, 2019), *phangorn* (Schliep, 2011), and *phytools* (Revell, 2012) in *R* version 4.2.3 (R Core Team, 2023). Using fossil calibrations at two nodes, Enarthronota separated from the other oribatid mite taxa (326–336 mya; Subías & Arillo, 2002) and Nothridae separated from the other higher taxa (99–122 mya; Arillo et al., 2016), we converted the phylogram to an ultrametric chronogram, using the strict clock model to transform branch lengths. For the remaining oribatid mite taxa without an 18S sequence, we grafted them onto the chronogram based on their taxonomic affiliation using the *www.add.species.to.genus()* function in the *R* package *phytools* (Figure S3).

### 2.3 Trait data collection

We collected a total of 18 traits for the 57 oribatid mite species from literature (Maraun et al., 2019, 2023; Weigmann, 2006). The traits included ecological traits (α‐niche traits; Maraun et al., 2019, 2023) and morphological characters (β‐niche traits; Weigmann, 2006; Table 1). We included a large number of traits thereby to select those with the most pronounced responses, even though little is known on their functionality. For oribatid mites determined only at genus level, we randomly assigned a trait state to the genus (Data S1).

**TABLE 1:** List of traits examined with corresponding weights assigned to the Gower distance matrix.

Trait type	Trait	Presumed function	Pre‐weight	Post‐weight
**α‐niche**	**Reproductive mode**	**Population growth, dispersal**	**0.083**	**0.152**
	**Trophic guild**	**Indicating food source**	**0.103**	**0.068**
**β‐niche**	**Length**	**Habitat adaptation**	**0.060**	**0.241**
	**Width**	**Habitat adaptation**	**0.076**	**0.200**
	Body form (length‐to‐width ratio)	Habitat adaptation, e.g., soil pore space	0.048	excluded
	**Number of claws**	**Habitat adaptation, resource acquisition**	**0.052**	**0.017**
	Area porosae	Chemical perception	0.043	excluded
	Sacculi	Chemical perception	0.049	excluded
	Notogaster structure		0.032	excluded
	Notogaster setae	Protection	0.049	excluded
	**Concealability**	**Protection from enemies**	**0.065**	**0.110**
	**Prodorsum structure** (lamellae, costulae)	**Protection from enemies**	**0.083**	**0.137**
	Dirt (debris attached to notogaster surface)	Protection, camouflage	0.026	excluded
	**Sclerotization**	**Desiccation protection**	**0.097**	**0.075**
	Sensillus setae	Perception	0.030	excluded
	Sensillus rami	Perception	0.034	excluded
	Sensillus shape		0.034	excluded
	Mouthparts	Feeding	0.034	excluded

*Note*: Traits with preweight <0.05 were excluded from further analyses. The weights of the eight traits (in bold) used to calculate the three distance matrices representing total, α‐ and β‐niche dissimilarity are given in the post‐weight column. See Data S1 for trait states and values.

### 2.4 Phylogenetic and trait distances of communities

We used distance‐based metrics, mean pairwise distance (MPD), and mean nearest taxon distance (MNTD), to quantify phylogenetic and trait distances between pairs of species in a community. We transformed the chronogram into a phylogenetic distance matrix using cophenetic distances and calculated the abundance‐weighted phylogenetic MPD for each community using the *mpd()* function in the *picante* package (Kembel et al., 2010). For trait dissimilarity between species, we scaled continuous trait variables (i.e., body length, width, and shape) and set the α‐ and β‐niche traits as groups. We calculated pairwise distances using all traits with the balanced Gower method, with w‐type set to “optimized” in the *gawdis* function (de Bello et al., 2021; Gower, 1971). The weights of each trait are given in Table 1 (preweight). We then retained the traits with a weight >0.05 and finally included eight traits (two for α‐niche and six for β‐niche traits) to represent the total trait dissimilarity between species (Table 1; post‐weight). The α‐niche traits included reproductive mode and trophic guild, and the β‐niche traits included body length and width, prodorsum structure, concealability, sclerotization, and number of claws. We constructed the α‐ and β‐niche distance matrices with the w‐type set to “analytic” in the *gawdis()* function and visualized the three trait distance matrices (i.e., total, α‐ and β‐niche dissimilarity) with dendrograms using the *ggtree* (Yu, 2020) and *ggnewscale* packages (Campitelli, 2023) (Figures S4–S6). For each community, we calculated the abundance‐weighted MPD for β‐niche traits and MNTD for α‐niche traits, because MPD quantifies the average dissimilarity of all species pairs and is more sensitive to environmental filtering, whereas MNTD only averages distances between nearest relatives and is more sensitive to identifying biotic interactions such as competition or resource‐based niche partitioning (Swenson, 2014, 2019).

### 2.5 Statistical analysis

To test the first hypothesis, we used the observed trait and phylogenetic MPDs as response variables in a linear mixed‐effects model using the *lme4* package (Bates et al., 2015). We included monoculture/mixed forest as a fixed factor and site as a random factor. To test the effect of monoculture/mixed forest on oribatid mite phylogenetic or trait diversity, we performed a Wald Chi^2^ test using the *car* package (Fox & Weisberg, 2019) and visualized the results using *ggplot2* (Wickham, 2016) and *gridExtra* (Auguie, 2017).

To test the second and third hypotheses on community assembly processes, we calculated standardized effect sizes (SES) of MPD (β‐niche traits) and MNTD (α‐niche traits) with the “richness” null model and 999 randomizations, where species were randomly drawn from the species pool, while keeping the species richness of the local community constant. We assessed the effects of monoculture/mixed forest and tree identity on oribatid mite trait dissimilarity by comparing five linear mixed effects models: a null model with site as a random effect, a model with monoculture/mixed forest as a fixed factor and site as a random effect, and three models with the presence of Douglas fir, Norway spruce or beech as a fixed factor and site as a random effect. We determined the best model using the lowest Akaike Information Criterion corrected for small sample size and then assessed model fit with the Wald Chi^2^ test. We visualized clustering or overdispersion patterns with boxplots and validated significance with t‐tests against 0, which indicates random assembly processes. We used paired t‐tests to test for differences between the SES of the α‐niche trait MNTD and the SES of the β‐niche trait MPD for the same communities.

We examined the phylogenetic signal of the α‐ and β‐niche dimensions. Given that different traits are intercorrelated, we performed a principal coordinate analysis (PCoA) on each of the α‐ and β‐niche distance matrices using the *ape* package (Paradis & Schliep, 2019). We used Blomberg's K to quantify the phylogenetic signal in species scores on the first PCoA axis using the *phylosig()* function in the *phytools* package (Blomberg et al., 2003; Revell, 2012) and randomized species scores to test the signal model against the white noise (i.e., no phylogenetic signal) model. A Blomberg's K equal to 1 suggests that the evolution of niche space is consistent with the expectation of a Brownian motion model, and that trait variation between species can be predicted by phylogeny.

## 3 RESULTS

### 3.1 Phylogenetic and trait diversity of oribatid mites

Phylogenetic MPD of oribatid mite communities was slightly but not significantly higher in mixed forests than in monocultures (Chi^2^ = 0.52, *p* > .05). By contrast, their trait MPD was significantly higher in monocultures than in mixed forests (Chi^2^ = 4.36, *p* = .036; Figure S7).

### 3.2 Community assembly processes revealed by α‐ and β‐niche traits

The best model for oribatid mite community trait dissimilarity was the one including beech as a fixed factor and site as a random effect for both the α‐niche trait MNTD (Chi^2^ = 9.03, *p* = .003) and the β‐niche trait MPD (Chi^2^ = 6.73, *p* = .009; Table 2). However, the model including Douglas fir was the least supported of the five models for both the α‐ and β‐niche traits. The model including monoculture/mixed forest was the second best model for both the α‐niche (Chi^2^ = 4.14, *p* = .042) and β‐niche traits (Chi^2^ = 7.21, *p* = .007), marginally better than the null model with only site as a random effect (Table 2).

**TABLE 2:** Support of linear mixed effects models for trait dissimilarity (MNTD: standardized effect size of mean nearest taxon distance; MPD: standardized effect size of mean pairwise distance) of oribatid mite communities as predicted by monoculture/mixed forest and tree identity (species presence).

Trait parameter	Model	AICc
α‐niche trait MNTD	~ 1 + (1|site)	123.3
	~ Monoculture/mixed forest + (1|site)	122.1
	~ Douglas fir + (1|site)	126.1
	~ Norway spruce + (1|site)	125.1
	~ European beech + (1|site)	**118.1**
β‐niche trait MPD	~ 1 + (1|site)	132.0
	~ Monoculture/mixed forest + (1|site)	130.6
	~ Douglas fir + (1|site)	134.6
	~ Norway spruce + (1|site)	134.0
	~ European beech + (1|site)	**128.3**

*Note*: The Akaike Information Criterion corrected for small sample size (AICc) of the best model is indicated in bold.

**FIGURE 1:**
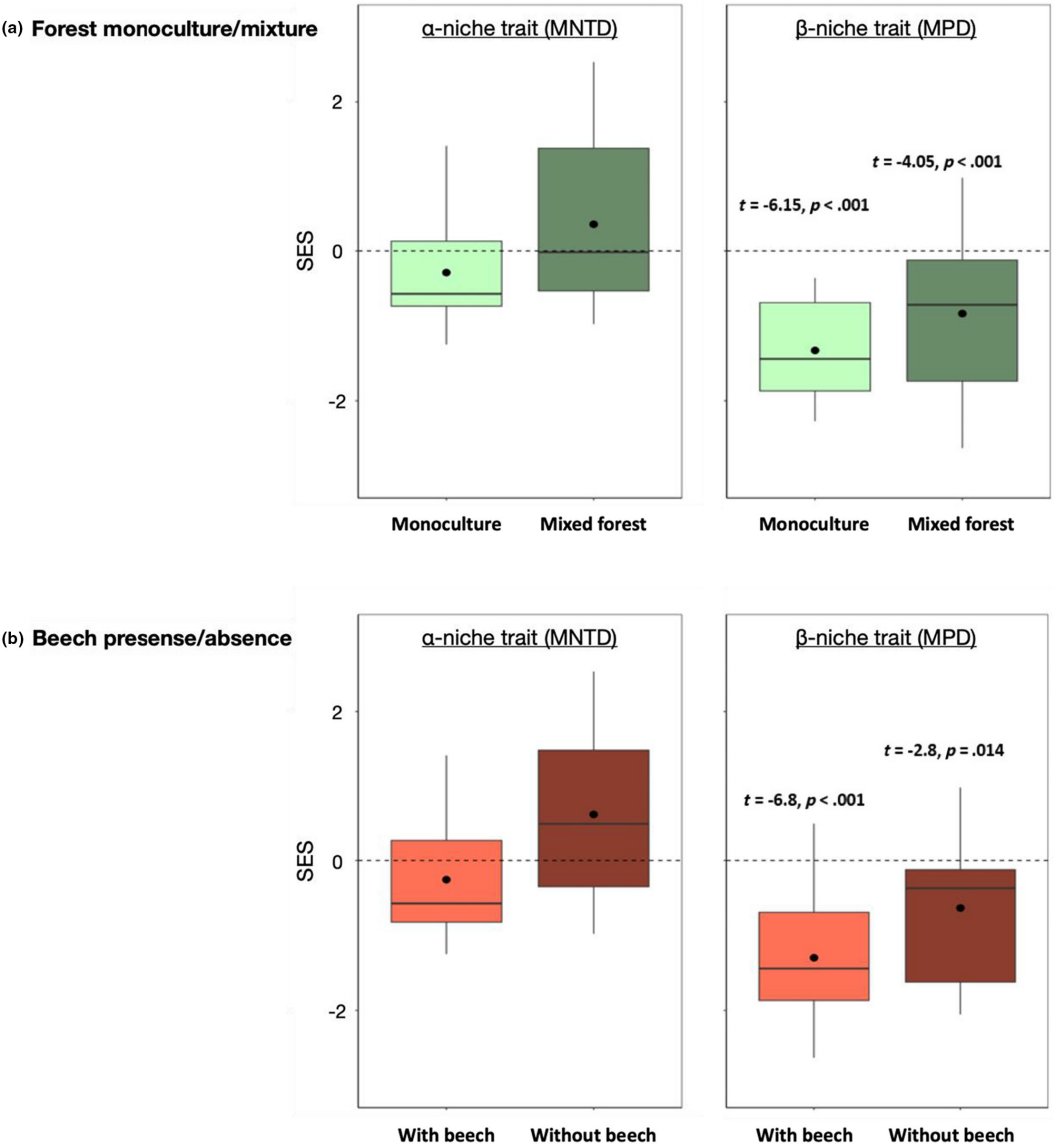
Standardized effect size (SES) of mean nearest taxon distance (MNTD) of the α‐niche traits (left panel) and mean pairwise distance (MPD) of the β‐niche traits (right panel) of oribatid mite communities in monocultures or mixed forests (a) and in forests with or without European beech trees (b). The black line in boxplots represents the median, the black dot the mean and the whiskers the minimum and maximum values. The dashed line represents the trait dissimilarity between co‐occurring species expected from random community assembly.

Oribatid mite species differed more in their α‐niche than in their β‐niche in monocultures (mean difference of SES = 1.19, *t* = 4.6, *p* < .001) and mixed forests (mean difference of SES = 1.35, *t* = 4.1, *p* < .001), as well as in forests with beech (mean difference of SES = 1.25, *t* = 5.1, *p* < .001) and without beech (mean difference of SES = 1.26, *t* = 3.5, *p* = .003; Figure 1). There was no significant pattern of overdispersion of α‐niche traits compared to the random assembly expectation, although the mean SES values of α‐niche MNTD were negative in monocultures but positive in mixed forests (Figure 1a), and negative in forests with beech but positive in forests without beech (Figure 1b). The β‐niche of oribatid mite communities generally showed clustering patterns that were more significantly different from the random assembly expectation in monocultures (*t* = −6.15, *p* < .001) than in mixed forests (*t* = −4.05, *p* < .001; Figure 1a) and in forests with beech (*t* = −6.8, *p* < .001) than in forests without beech (*t* = −2.8, *p* = .014; Figure 1b).

### 3.3 Evolutionary patterns of α‐ and β‐niches

Patterns of phylogenetic signal differed between α‐ and β‐niche traits; for α‐niche traits, the K‐value was not significantly different from a white noise model (*K* = 0.087, *p* > .05), whereas β‐niche traits showed phylogenetic signal with a K‐value significantly higher than predicted by the white noise model (*K* = 1.25, *p* < .001).

## 4 DISCUSSION

### 4.1 Phylogenetic and trait diversity of oribatid mites in monocultures and mixed forests

We hypothesized that both phylogenetic diversity and trait diversity of oribatid mites are higher in mixed forests than in monocultures, but the results argue against this hypothesis. Trait MPD was significantly higher in monocultures than in mixed forests and phylogenetic MPD was only slightly higher in mixed forests than in monocultures. From a functional perspective, high trait diversity in communities indicates different contribution of taxa to ecosystem processes, which may promote ecosystem resilience (Laureto et al., 2015). Thus, higher functional diversity of oribatid mites in forest monocultures suggests that oribatid mites are more likely to contribute to ecosystem processes in monocultures than in mixed forests (Behan‐Pelletier, 1999; Moore et al., 1988; Pande & Berthet, 1973). Alternatively, the investigated traits are more closely associated to the functions in forest monocultures. However, functional redundancy has been proposed to be common among soil animal species (Magilton et al., 2019), and the selected oribatid mite traits may not be considered functional in the sense of effect traits involved in ecosystem processes, but they may be functional in terms of individual fitness and hence response traits.

The different patterns of phylogenetic diversity and trait diversity highlight the importance of a comprehensive, integrative approach that considers both phylogeny and traits when assessing biodiversity and suggest that phylogenetic and functional dimensions provide complementary information to the commonly used taxonomic diversity indices. Our results also suggest that using phylogeny as a proxy for trait or functional diversity may be misleading, as previously suggested (Swenson, 2019). Alternatively, integrating species pool phylogeny into community analyses provides insight into evolutionary processes relevant to community assembly of soil animals, as demonstrated for isotomid springtails (Xie et al., 2022): The divergence of species traits in the past is a prerequisite for their current assembly. The species pool of oribatid mites in our study included a large number of derived lineages. The phylogeny can serve as a backbone for studying the evolution of oribatid mite traits as related to environmental factors such as soil pH and moisture in different forest types, similar to the aforementioned study.

### 4.2 Community assembly processes revealed by different niche dimensions

In support of our second hypothesis, patterns of overdispersion and clustering occurred simultaneously within oribatid mite communities, with community assembly driven by filtering processes as indicated by β‐niche traits. Within local communities, α‐niche traits differed more than β‐niche traits, but α‐niche traits were clustered in some communities in beech forests or mixed forests with beech, suggesting that environmental filtering is more important than resource‐based niche partitioning at these sites. This contrasts with the results of the small‐scale study by Magilton et al. (2019), which examined trophic traits of oribatid mites in deciduous forests and showed that resource‐based niche partitioning plays an important role in structuring oribatid mite communities. However, beech was not included in their study, pointing to the importance of tree identity in the assembly of soil animal communities and suggesting that litter quality may act as a filter in the assembly of oribatid mite species. Overall, the results suggest that resource‐based niche partitioning and environmental filtering processes operate via different traits in the assembly of oribatid mite communities, and their relative importance varies with tree identity.

In addition to environmental filtering and niche‐based processes, oribatid mite species could have been assembled by stochastic processes, as proposed by the neutral theory of ecology and evolution (Hubbell, 2006). A number of studies have investigated the relative contribution of deterministic and stochastic processes in structuring oribatid mite communities, with somewhat conflicting results, but concluded that both processes occur simultaneously (Caruso et al., 2012; Maaß et al., 2015). Further trait‐based studies are needed to determine the drivers of soil animal diversity and their community assembly processes, for example by considering larger environmental or geographic gradients combined with a wider range of traits of different niche dimensions (Winemiller et al., 2015).

Tree identity explained trait dissimilarity of oribatid mite communities better than forest type (monocultures vs. mixed forests), supporting our third hypothesis. Patterns were most pronounced when beech trees were present, both in monocultures and mixed forests. The α‐niche traits of oribatid mites were filtered, suggesting that beech litter is a poor resource for oribatid mites (Pollierer et al., 2009; Potapov et al., 2019). However, it may also point to the role of predators in driving community assembly as a filter, reflected in environmental tolerance traits (β‐niche) such as sclerotization and concealability. In contrast to beech, the lack of significant patterns in trait dissimilarity of oribatid mites in Douglas fir forests suggests the dominance of neutral assembly processes. Litter quality may therefore only be important for certain species (e.g., endophagous taxa such as *Phthiracarus* spp. or *Microtritia minima*), whereas other species, such as those at high trophic positions (e.g., Oppiidae, Suctobelbidae and Quadroppiidae) may suffer from reduced root‐derived resources in Douglas fir stands (Lu, 2021; Lu, Cordes, et al., 2022). As the patterns of trait dissimilarity were similar for the monoculture/mixed forest fixed factor model and the beech presence model, the strong filtering effect in mixed forests was likely due to the effect of beech and not mixed forests in general.

### 4.3 Evolutionary patterns of α‐ and β‐niche space

Analysis of the phylogenetic signal of α‐ and β‐niche traits indicated convergent evolution for the former but conserved evolution for the latter, consistent with previous findings (Schaefer & Caruso, 2019). Using the concept of α‐ and β‐niche traits for studying Collembola fatty acids also indicated that β‐niche traits (i.e., fatty acids related to physiology rather than resources) are phylogenetically conserved, whereas α‐niche traits (i.e., fatty acids related to resources) are generally labile (Chen et al., 2017). The similarity of patterns to previous studies supports this classification as generally applicable to soil organisms, and our approach of using different distance measures for different niche dimensions, with MNTD indicating resource‐based niche partitioning and MPD providing information on historical environmental constraints in morphological traits.

## 5 CONCLUSIONS

Our study provided insight into the mechanisms of oribatid mite community assembly in forest soils with respect to different niche dimensions. We have shown that different traits of oribatid mites contribute differently to community assembly processes in forests with native European beech compared to forests with only conifers, especially those with non‐native Douglas fir. This highlights the importance of tree identity in structuring oribatid mite communities, with a complex interplay between environmental filtering, resource‐based niche partitioning and stochastic processes in shaping species composition. Our results also shed light on effects of silvicultural practices on the phylogenetic and functional diversity of decomposer communities in forest ecosystems, calling for a comprehensive approach that considers different niche dimensions when assessing biodiversity. Our study challenges the notion of using phylogeny as a sole proxy for trait or functional diversity and highlights the need for integrative analyses that capture the evolutionary processes underlying community assembly. Further research integrating larger environmental gradients and a wider range of traits will be essential to unravel the drivers of soil animal diversity and community assembly processes.

## AUTHOR CONTRIBUTIONS

**Johanna Elisabeth Noske:** Conceptualization (lead); data curation (equal); formal analysis (equal); investigation (lead); methodology (lead); project administration (supporting); software (equal); validation (equal); visualization (lead); writing – original draft (lead); writing – review and editing (equal). **Jing‐Zhong Lu:** Data curation (equal); formal analysis (equal); investigation (lead); project administration (supporting); writing – review and editing (equal). **Ina Schaefer:** Conceptualization (supporting); resources (equal); software (equal); supervision (supporting); writing – review and editing (equal). **Mark Maraun:** Conceptualization (equal); writing – review and editing (equal). **Stefan Scheu:** Conceptualization (supporting); funding acquisition (lead); methodology (supporting); project administration (lead); resources (equal); supervision (supporting); validation (supporting); writing – original draft (supporting); writing – review and editing (equal). **Ting‐Wen Chen:** Conceptualization (lead); data curation (supporting); formal analysis (equal); investigation (supporting); methodology (lead); supervision (lead); validation (equal); visualization (supporting); writing – original draft (lead); writing – review and editing (equal).

## FUNDING INFORMATION

This work was supported by the German Research Foundation through the Research Training Group 2300: “Enrichment of European beech forests with conifers: impacts of functional traits on ecosystem functioning” (Grant ID: 316045089). Open Access funding enabled and organized by Projekt DEAL.

## CONFLICT OF INTEREST STATEMENT

The authors have no conflicts of interest to declare.

## Supporting information


Data S1.

## Data Availability

Data and codes used in the study are available in the Supplementary Material. Community data are available from Lu, Bluhm, et al. (2022) (https://doi.org/10.1594/PANGAEA.944676) or on request from co‐author Jing‐Zhong Lu (jlu@gwdg.de).
