# Supplementary material for: Niche dimensions in soil oribatid mite community assembly under native and introduced tree species
Source: Ecol Evol. 2024 May 20;14(5):e11431. doi: 10.1002/ece3.11431 (PMC11103279; doi:10.1002/ece3.11431)
Supplement: Supplementary file 1 — Data S1. [file ECE3-14-e11431-s001.zip › Noske_MA_paper_supp_20240206.docx]

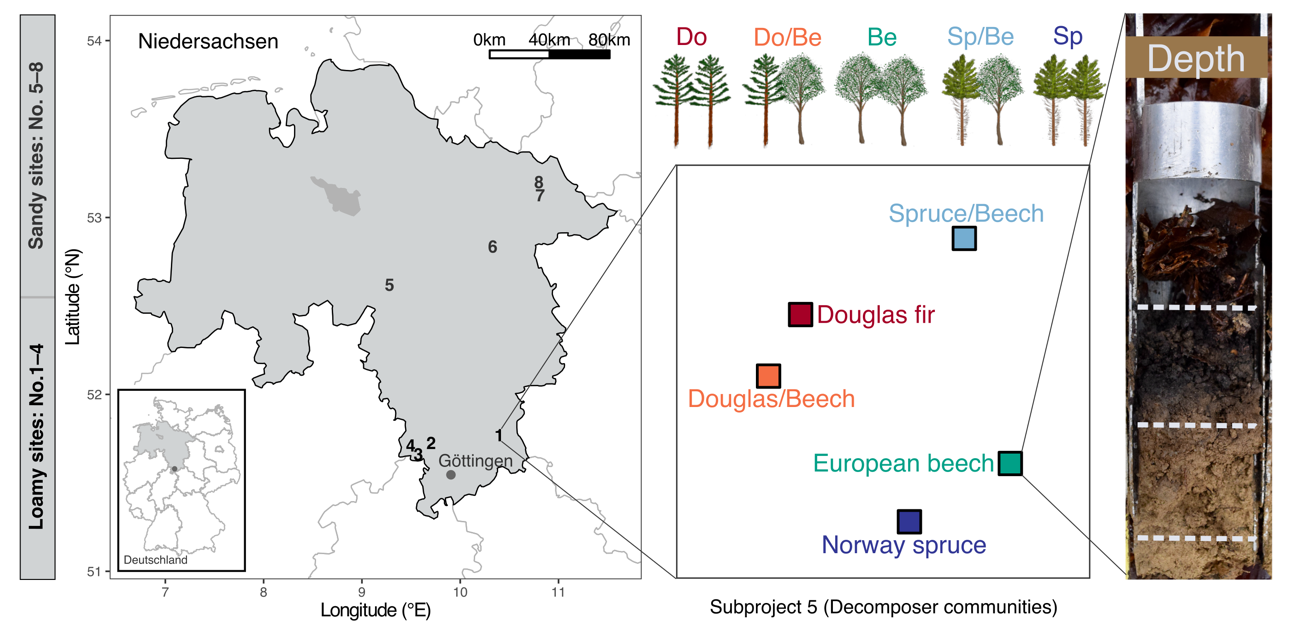


**Fig. S1** Study sites of the EnriCo project in Lower Saxony, Germany (tree drawings: Alice Penanhoat; adapted from Lu et al. 2022).

Lu, Jing-Zhong; Cordes, Peter Hans; Maraun, Mark; Scheu, Stefan (2022): High consistency of trophic niches in generalist arthropod species (Oribatida, Acari) across soil depth and forest type. In *Ecology and evolution* 12 (12), e9572. DOI: 10.1002/ece3.9572.


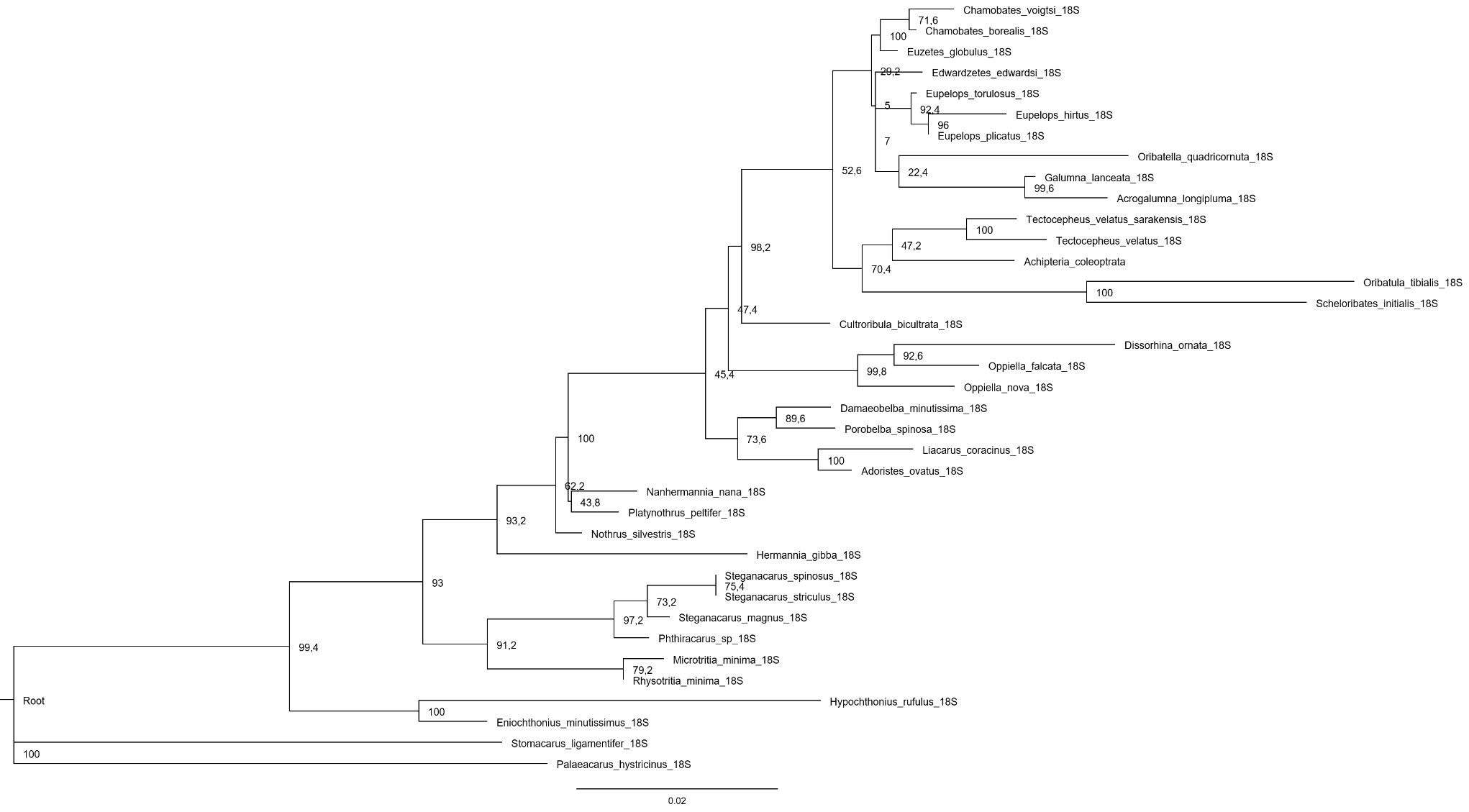


**Fig. S2** Molecular phylogeny (maximum likelihood phylogram with 500 bootstraps) of oribatid mites from the EnriCo sites (Lower Saxony, Germany). Palaeacarus hystricinus and Stomacarus ligamentifer were used as outgroups.


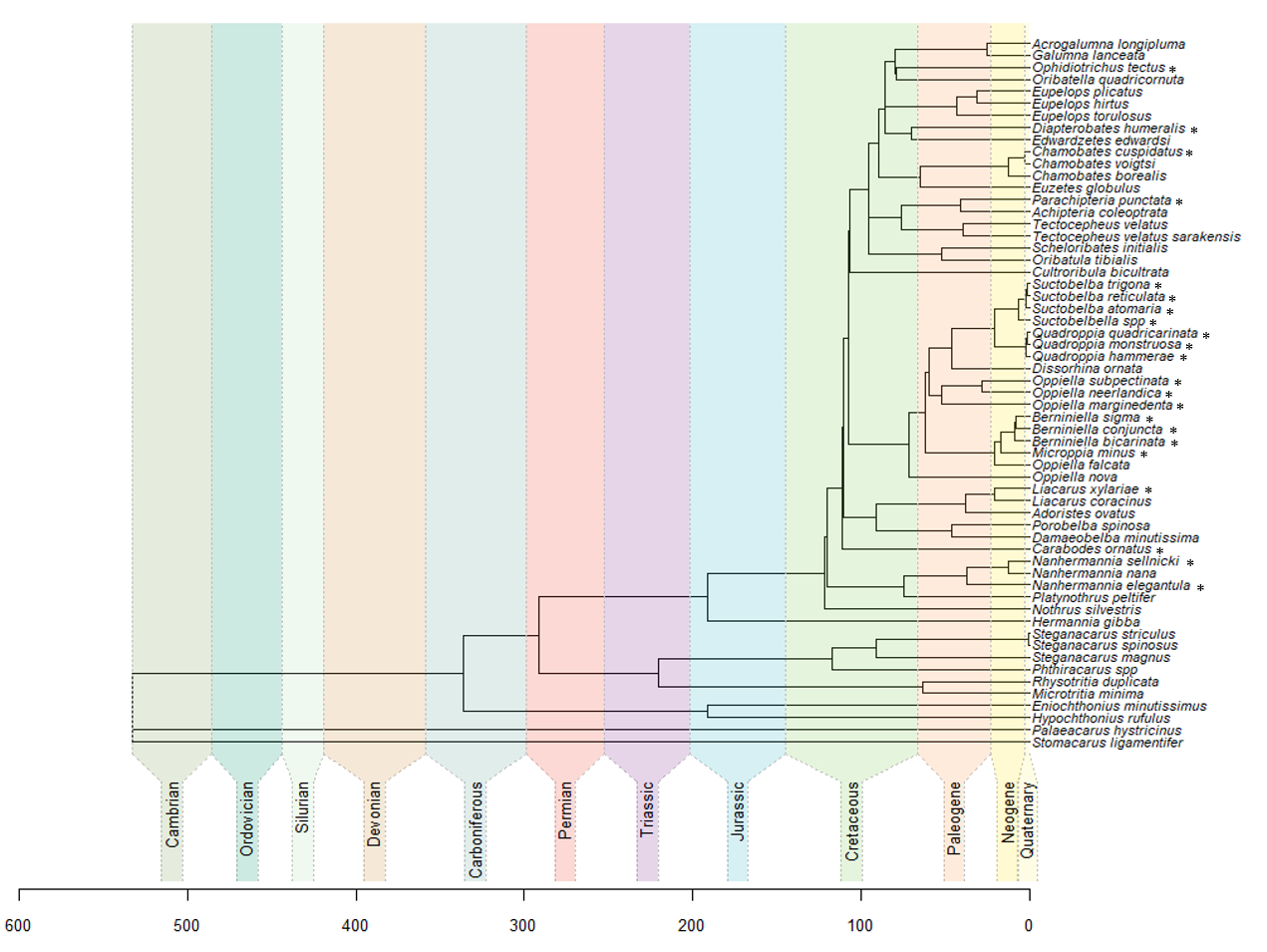


**Fig. S3** Phylogeny (chronogram) of oribatid mites from the EnriCo sites (Lower Saxony, Germany). *Palaeacarus hystricinus* and *Stomacarus ligamentifer* were used as outgroups. Species marked with * were added based on taxonomic affiliation.


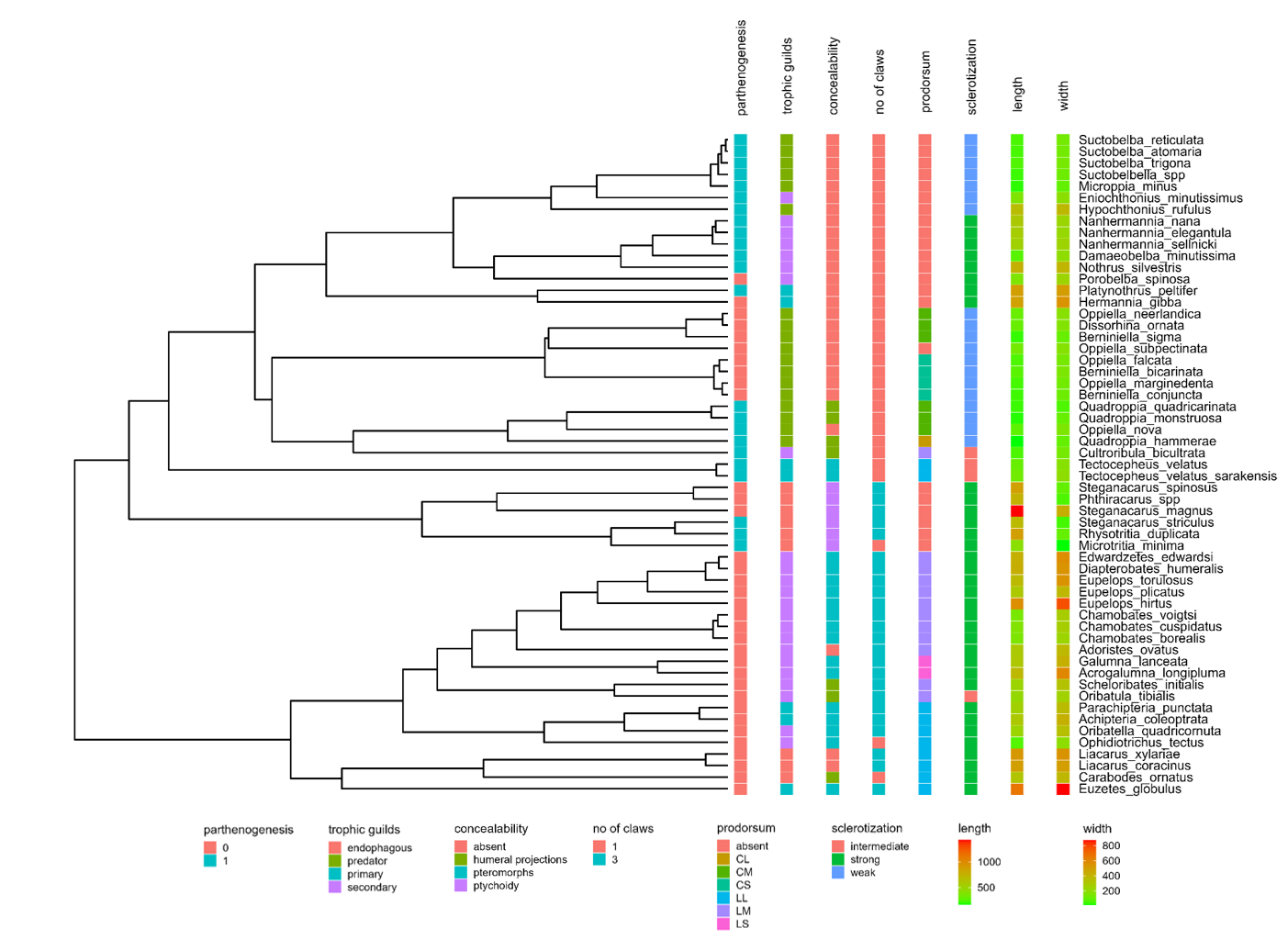


**Fig. S4** Trait dendrogram of oribatid mites generated by the eight traits. For an explanation of abbreviations, see Table 1 and supplementary data.


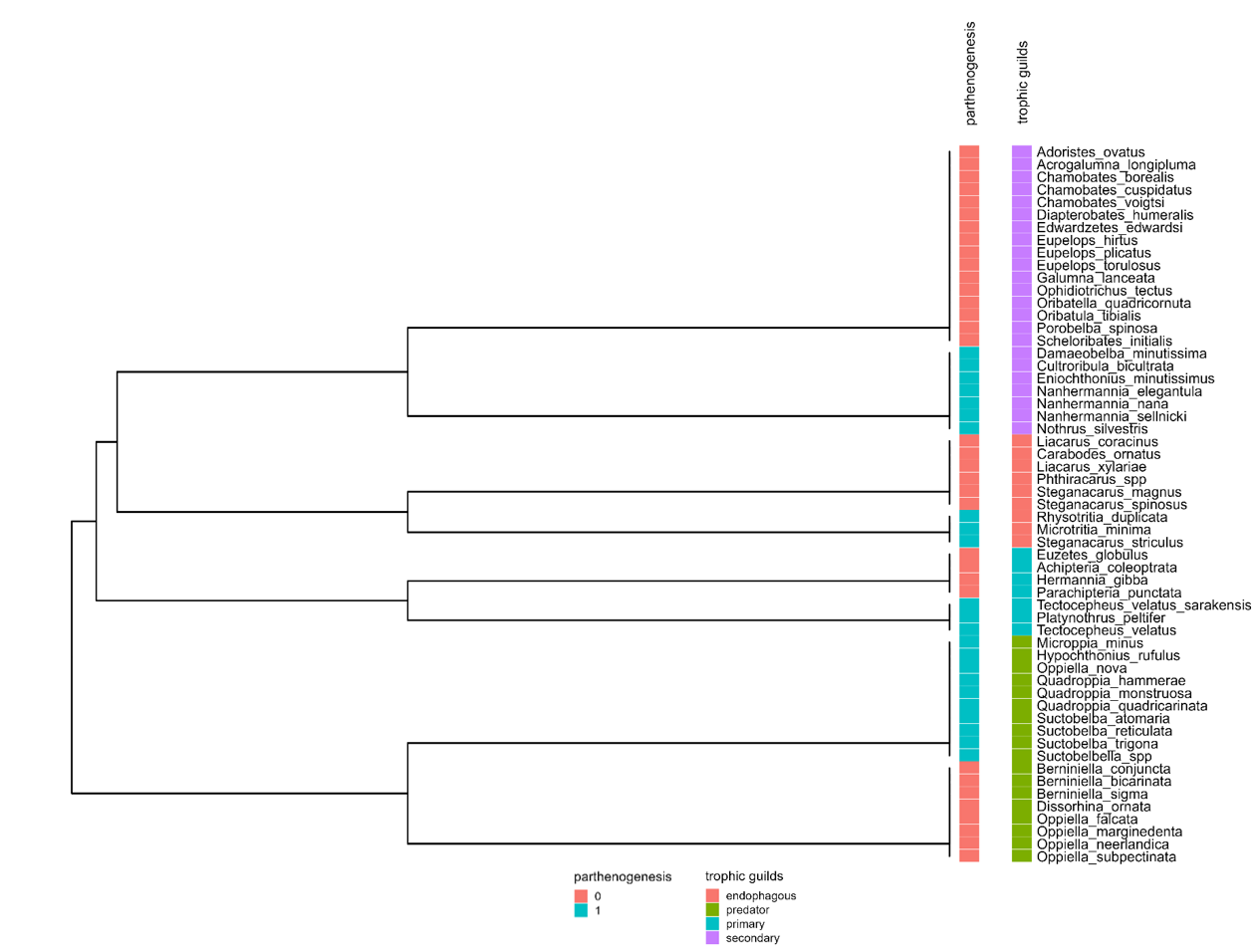


**Fig. S5** Trait dendrogram of oribatid mites generated by the α-niche traits.


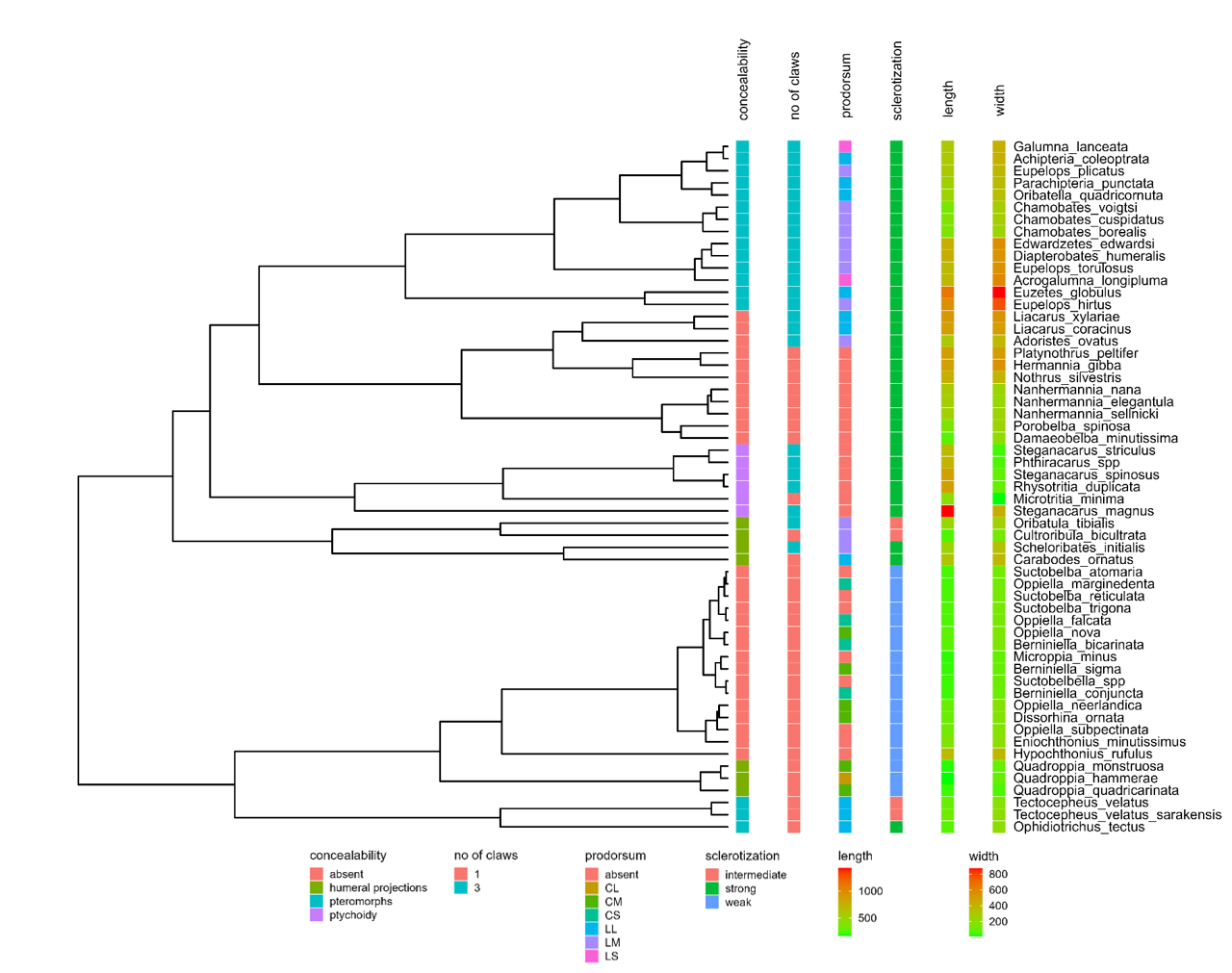


**Fig. S6** Trait dendrogram of oribatid mites generated by the β-niche traits. For an explanation of abbreviations, see Table 1 and supplementary data.


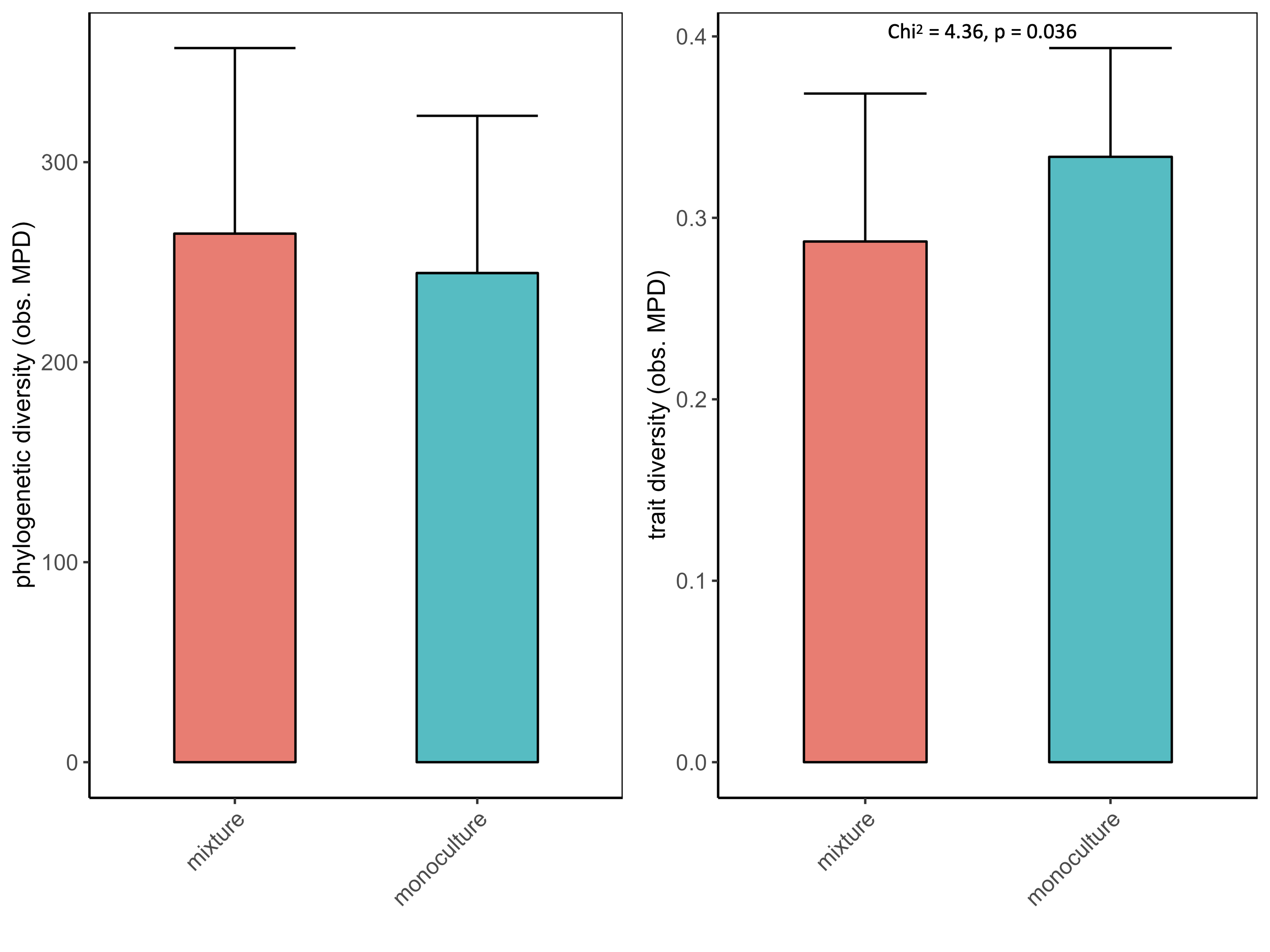


**Fig. S7** Phylogenetic diversity (left) and trait diversity (right) measured by mean pairwise distance (obs.MPD) in forest mixtures and monocultures. Bars indicate mean values; whiskers indicate standard deviation.
